# Genomic Perspective on Mouse Liver Cancer Models

**DOI:** 10.3390/cancers11111648

**Published:** 2019-10-25

**Authors:** Sun Young Yim, Ju-Seog Lee

**Affiliations:** 1 Department of Internal Medicine, Division of Gastroenterology and Hepatology, Korea University College of Medicine, Seoul 136-701, Korea; eug203@naver.com; 2 Department of Systems Biology, Department of Cancer Biology, The University of Texas MD Anderson Cancer Center, Houston, TX 77030, USA

**Keywords:** mouse model, hepatocellular carcinoma, ctnnb1, arid1a, sv40, genomics, hippo pathway, genomic resemblance

## Abstract

Selecting the most appropriate mouse model that best recapitulates human hepatocellular carcinoma (HCC) allows translation of preclinical mouse studies into clinical studies. In the era of cancer genomics, comprehensive and integrative analysis of the human HCC genome has allowed categorization of HCC according to molecular subtypes. Despite the variety of mouse models that are available for preclinical research, there is a lack of evidence for mouse models that closely resemble human HCC. Therefore, it is necessary to identify the accurate mouse models that represent human HCC based on molecular subtype as well as histologic aggressiveness. In this review, we summarize the mouse models integrated with human HCC genomic data to provide information regarding the models that recapitulates the distinct aspect of HCC biology and prognosis based on molecular subtypes.

## 1. Introduction

Hepatocellular carcinoma (HCC) represents 75% of cases of primary liver cancer [1] and has worldwide prevalence of 841,000 cases per year, making it the sixth most common cancer globally, and 782,000 deaths occur annually [2]. With similar mortality to prevalence rates, hepatocellular carcinoma (HCC) ranks fifth in terms of global cases and second in terms of deaths in males. Given the international burden of disease, implementation of surveillance programs for at risk population have been implemented. However, despite the effort to detect an asymptomatic or subclinical HCC, 30–60% of HCC tumors are detected at an advanced stage [3] resulting in a dismal prognosis [1]. In addition, limited systemic treatment options (first-line: sorafenib, lenvatinib and second-line: regorafenib, nivolumab) requires improvement in treatment and much remains to be discovered in clinical and experimental studies. 

More than 90% of HCCs arise in the context of chronic hepatitis and cirrhosis. Hepatocarcinogenesis is a complex and multistep process involving the accumulation of genetic changes and resulting in altered expression of cancer-related genes, such as oncogenes and tumor suppressor genes, and their related molecular signaling pathways. Furthermore, HCC genetic aberrations show substantial heterogeneity reflecting effects of etiology, ethnicity, and environmental exposures. Strategies to understand such complex process and improve poor survival of HCC patients rely on good experimental models that accurately recapitulate the steps of hepatocarcinogenesis in human. 

The complex links between different risk factors and HCC development were poorly understood until the advancement in genomic studies of human HCC which enabled systemic analysis of genomic data providing insight into the biology and pathogenesis of HCC. Currently, genomic studies of human HCC are progressing, and systematic analysis of genomic data is providing insight into the biology and pathogenesis of HCC. Recent studies attempted to classify HCC based on genetic, transcriptional, methylation, or miRNA levels [4,5] and many HCC genome studies have catalogued potential driver genes in HCCs [6,7]. Many efforts were made to identify molecularly distinct subtypes of HCC with different clinical outcomes. Recently, comprehensive integrative genomic analysis of HCC has delineated the HCC genomic landscape with *TERT* being the mostly repressed in somatic cells [5]. *TERT* promoters have been found to be mutated in more than 50% of HCC tissue samples examined, making them the most frequently occurring single-nucleotide mutations observed in HCC [8]. Other frequent mutations are *CTNNB1*, *TP53*, *ARID1A*, and *AXIN1*. 

The laboratory mouse is one of the most preferred experimental systems for both basic and translational study of HCC due to the physiological, molecular, and genetic similarities to humans, the small size, large number of offspring, short lifespan, and low cost. Mice are invaluable tools in uncovering the molecular mechanisms of hepatocarcinogenesis by introducing genetic alterations observed from human HCC [5,9,10,11]. However, despite detailed characterization of genomic alteration in human HCC, genetic makeup of full-blown HCC mouse models is generally unknown and therefore it is unclear which mouse models are molecularly similar to human HCCs. For research purpose, such as evaluation of molecular target therapy and biomarker studies, the ideal mouse model should reproduce human HCC genetically, physiologically, and pathologically [12]. Although some mouse models have been proposed [13,14], most do not replicate human HCC and they are poorly characterized molecularly. Recently, we have identified the most relevant mouse models to human liver by integrating eleven different genomic signatures of human HCC to nine different HCC mouse models [15]. The present article will review common types of mice that are used for experimental studies with specific focus on molecular resemblance of mouse model to human HCC.

## 2. Conventional Mouse Models for Liver Cancer

**Figure 1:**
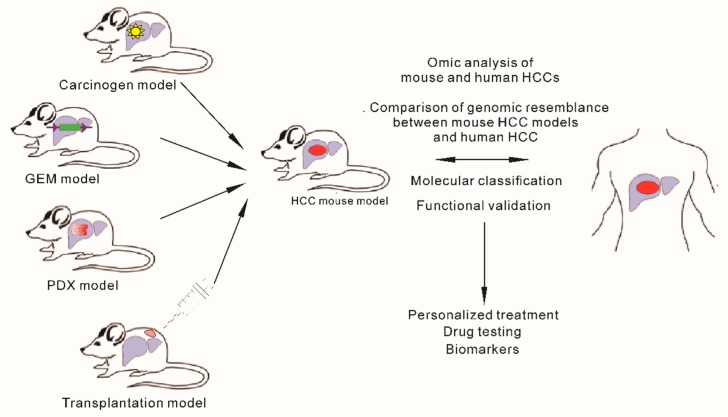
Schematic diagram of mouse models commonly used to develop hepatocellular carcinoma and integration of human and mouse genomics to select appropriate model for clinical therapeutic purpose.

A relatively large number of mouse models are presently available for the study of hepatocarcinogenesis. The common ones are chemically induced models, genetically engineered mouse (GEM) models, implantation models, and viral models. Our aim here is not to review all mouse models for liver cancer but rather to provide examples of the representative ones, mostly chemically induced or genetically engineered models (GEMs) that have been integrated to genomics of human HCCs (Figure 1). 

### 2.1. Chemically Induced Models 

Chemically induced hepatocarcinogenesis is caused by an irreversible process of structural DNA changes [16]. The most widely used chemical to induce liver cancer in mice is diethylnitrosamine (DEN) (Table 1). DEN is a DNA alkylating agent and when injected into juvenile mice, it forms mutagenic DNA adducts, which are generated rapidly in centrilobular hepatocytes [17,18]. DEN is metabolic activated in hepatocytes by enzymes of the cytochrome P450 family and acts as a complete carcinogen if injected into young mice younger than two weeks old with actively proliferating hepatocytes resulting in dysplastic nodules, which progress to carcinoma. If given to an older mouse, additional stimulation is required for instance, phenobarbital (PB), carbon tetrachloride, or high-fat diet feeding [19]. In addition, oxidative stress induced by reactive oxygen species (ROS) during DEN metabolization is known to contribute to hepatocarcinogenesis as they cause DNA, protein, and lipid damage [20]. 

The mutational landscape of a DEN-induced tumor was described in a recent study using the whole exome sequencing technique. A high burden of somatic mutation was observed and almost all of the DNA changes in the DEN-induced tumors were single-base substitutions [18]. Four recurrently mutated genes that are putative oncogenic drivers of DEN-induced tumors were *Hras*, *Braf*, *Egfr*, and *Apc*. The incidence of *Hras* was the highest reaching approximately 80% suggesting selective advantage during hepatocarcinogenesis. *Hras*, *Braf*, and *Egfr* mutations were present in every DEN-induced HCC with mutual exclusivity and may replace each other in terms of oncogenic drivers [18,21]. Activation of the *Ras/Raf/MEK/ERK* signal transduction pathway was the hallmark feature in the DEN-induced mouse model which is rare in human HCC [18,21,22]. Of note, *TP53* and *CTNNB1*, the most frequently altered genes in human were never found in DEN-induced tumors. Although *ctnnb1* mutation is not observed in DEN-induced model, its mutation is known to be observed in a two-stage model, the DEN-initiated and PB-promoted protocol [23,24]. In addition, loss of *Apc* function, mutation and aberrant nuclear expression of β-catenin may disrupt the canonical Wnt/β-catenin pathway [18]. 

The Stelic Animal Model (STAM) demonstrates non-alcoholic steatohepatitis (NASH) progression resembling the disease in humans. Since NASH is evolving as a major cause of non-viral HCC and may account for a large proportion of HCC in developed countries in recent years, HCC arising from NASH is important [25]. The STAM model is created by combination of chemical (a single subcutaneous injection of 200 μg of streptozotocin at two days after birth) and dietary intervention (high-fat diet ad libitum four weeks after injection) in C57BL/6 mice. STAM mice manifest NASH at eight weeks, which progresses to fibrosis at 12 weeks, and eventually develops into HCC at a rate of nearly 100% in males [26]. The STAM model had pathway mutation rates comparable to those in humans for most pathways with more alterations in receptor tyrosine kinase (RTK) signaling and chromatin-modification genes. This model also showed low mutation rate of *TP53* but the cell-cycle pathway alteration rate was similar to human tumors. The limitation of this model is that the model does not show obesity or insulin resistance, which is the common characteristics of patients with NASH.

### 2.2. Genetically Engineered Mouse Models

Genetically engineered mouse (GEM) models recapitulate the multistep process of hepatocarcinogenesis with multiple genetic and epigenetic changes occurring along each stage of progression toward cancer formation [27]. They are highly useful for assessing the impacts of a driver oncogene alone or in combination with other driver oncogenes or tumor suppressors [28,29]. Recently, the Cancer Genome Atlas (TCGA) characterized the genomic landscape of HCC using large-scale multi-platform analysis of HCC, including evaluation of somatic mutations and copy number alterations [5]. Commonly altered pathways such as cell cycle pathway, RTK/RAS/PI3K, and WNT pathways were also identified and therefore, data generated from GEMs would be useful in developing novel therapies and be tested in the preclinical setting if they recapitulate human HCCs.

The *MYC* oncogene is known to be activated in more than 50% of human cancers by multiple mechanisms, and its activation is frequently associated with poor prognosis and unfavorable outcome [30]. *MYC* plays a central role in multiple oncogenic processes by regulating cell proliferation, apoptosis, and metabolism and has been a key therapeutic target for treatment of many cancers, including HCC. Despite the high incidence of *MYC* oncogene activation, directly targeting *MYC* as a therapeutic method has proven to be a challenge for many decades because of the undruggable nature of its molecular activity [31]. *MYC* is frequently amplified in HCC (around 20% of HCC according to the Cancer Genome Atlas (TCGA) study [5]. GEMs expressing *MYC* under an albumin promoter develop HCC after a long period of latency [32,33]. Co-activation of *E2f1* with *Myc* significantly accelerates HCC development [34]. Interestingly, a large number of HCC tumors in *Myc* and *E2f1/Myc* GEM models show activation of *Ctnnb1* [34], suggesting *Ctnnb1* might be an important interacting partner of *Myc* for the development and progression of HCC. *Myc* expression caused mild to severe hepatic dysplasia in young mice, followed by hepatic adenomas in mice over 15 months of age and activation of *Ctnnb1* lead to dysregulation of the signaling function of β-catenin causing hepatocarcinogenesis [35]. 

*TGFα* is one of ligands of *EGFR* and the TGFA–EGFR–RAS–MAPK signaling pathway is commonly upregulated in HCC [36]. GEMs expressing *Tgfα* under the inducible MT-1 promoter develop HCC [37]. As expected, co-activation of *Myc* with *Tgfα* significantly boosts neoplastic development compared with the expression of these isolated genes. Persistent proliferation of the hepatocytes as early as the first week of life continues and a neoplastic lesion is developed within 40 weeks of age [34]. Activation of *Ctnnb1,* which was frequently observed in liver tumors of the *Myc* model, was rare in *Myc*/*Tgfα* double transgenic mice but nuclear factor kappa-light-chain-enhancer of activated B cells (*NF-κB*)–induced survival signaling was activated [38]. 

The oncogene *CTNNB1* encodes β-catenin, which is a subunit of the cadherin protein complex on the cellular surface that acts as a signaling molecule in the *WNT* pathway [39]. Aberrant activation of β-catenin has been observed in 20–30% of HCC patients and is one of the most frequently mutated genes [5,7,40]. Mutations in β-catenin are almost mutually exclusive with mutations in *TP53* [6]. Intriguingly, in GEM mice, introduction of activated mutants of β-catenin or overexpression of wild-type β-catenin induces only hepatomegaly and does not form any neoplastic foci in the liver [41,42], suggesting that β-catenin alone is insufficient to initiate tumorigenesis in liver. However, follow-up studies in these GEM mice showed that β-catenin collaborates with other signaling pathways to contribute to hepatocarcinogenesis. The *ras* family genes encode small GTPase proteins that transduce signals from the transmembrane receptors to the nucleus [43]. Mutation in *Ras* proteins lead to defects in the GTPase activity and constitutive activation of the downstream signals. Although *RAS* mutations are rare in human liver tumors, *Hras* mutation is detected frequently in the spontaneous and carcinogen-induced mouse liver tumors [44]. β-catenin was shown to cooperate with activated *Hras* to induce hepatocarcinogenesis [45]. Mutations in both β-catenin and *Hras* cause HCC development at an incidence of 100%, supporting the notion that β-catenin activation is one of the hits that may be critical to the development of HCC, but that additional aberrations are necessary to initiate and promote tumorigenesis.

The SV40 T antigen induces oncogenic transformation of normal cells, including hepatocytes, by inactivating the tumor suppressor genes *p53* and *Rb* and interacting with a number of signaling proteins such as HSC70, CBP/p300, CUL7, IRS1, FBXW7, and BUB1 [46]. Inactivation of the two best-known tumor suppressors result in high proliferation and uncontrolled cell growth leading to hepatocarcinogenesis. In GEM mice expressing the SV40 T-antigen under hepatic promoters, HCC developed after a short period of latency (4–12 weeks) and metastasis to the lungs is frequently observed [47,48]. Since tumor progression is very rapid, this model is considered to have some differences from human HCC tumors, which progress more slowly.

The Hippo pathway was first discovered in *Drosophila* and is evolutionarily well conserved. The normal function of the Hippo pathway is to repress growth and when Hippo signaling is attenuated, tissue overgrowth occurs. Because the Hippo pathway is required for restricting cell growth and proliferation, as well as to induce programmed cell death, many members of the pathway are known to be involved in tumor development. *YAP1*, a component of the Hippo pathway acts as a potential oncogene in mouse HCC [49]. This oncogenic function of *YAP1* is further supported by tumor suppressors such as *Mst1/2* and *Sav1* which inhibit *YAP1* activity by phosphorylation [50]. As a result, *Mst1/2* and *Sav1* knockout (KO) in liver leads to the development of HCC [51,52]. Recent studies have shown that activation of *YAP1* and *TAZ* oncogenes in HCC is significantly associated with shorter survival rate, higher recurrence rate, and resistance to chemotherapy [51,53,54,55], indicating the importance of *YAP1/TAZ* in the development of HCC. 

The AT-rich interaction domain 1A (*ARID1A*) a subunit of a switch/sucrose-nonfermentable (SWI/SNF) chromatin remodeling complex is also frequently mutated in HCC (in up to 20% of cases). Most cancer-associated mutations in *ARID1A* appear to be loss-of-function mutations; nonsense or frameshift rather than missense mutations including HCC, suggesting that *ARID1A* is a tumor suppressor. An HCC mouse model for *ARID1A* has recently been generated but showed an unexpected and complicated phenotype. In contrast to the general notion of *ARID1A* as a tumor suppressor, as demonstrated in the colon cancer model [56], deletion of *Arid1a* in mouse liver protected against development of HCC [57], suggesting that *Arid1a* is necessary for the initiation of tumorigenesis in hepatocytes. The critical activity of *Arid1a* in tumor initiation appears to be related to its transcriptional regulation of the CYP450 family, which oxidizes metabolites and generates ROS in hepatocytes. High expression of *Arid1a* in hepatocytes promoted tumorigenesis by increased CYP450-mediated production of ROS. In contrast to its tumor-promoting activity during tumor initiation, deletion of *Arid1a* accelerated HCC tumor progression and metastasis in late stage of HCC development, further indicating the complicated roles for *Arid1a* in HCC development. Metastasis-suppressive activity is related to a decrease in global chromatin accessibility and reduced expression of genes that inhibit metastasis. In contrast, *Arid1a* deficiency promotes HCC progression at later stage by activating Ang2-dependent angiogenesis due to augmentation of histone H3K27ac modification at the *Ang2* gene locus [58]. Although these opposite roles of *Arid1a* at different stages of HCC development are interesting, it is not surprising to find that epigenetic regulators have highly context-specific functions as they play critical roles in remodeling chromatin that can support the actions of both oncogenic and tumor-suppressive networks.

Receptor tyrosine kinases (RTKs) have a role in tissue homeostasis and their oncogenic activation leads to cancer. The *Alb-R26^Met^* mouse was generated to explore in vivo vulnerability versus robustness to slight changes in non-oncogenic RTK levels [59]. A subtle increase in wild-type Met RTK levels in the liver developed spontaneous tumors which highlights the heightened vulnerability of liver cells to subtle changes in non-oncogenic RTK levels, allowing them to acquire a molecular profile that facilitates the full tumorigenic program. In addition, the regulatory networks underlying tumorigenesis analyzed using “educated guess” drug screen led to the identification of new, deleterious synthetic lethal interactions where synergistic effects of *Mek*, *Rsk*, and *Cdk1/2* in combination with *Bcl-XL* inhibition was observed on a panel of liver cancer cells [59]. The *Alb-R26^Met^* model was also characterized by hypermethylation of CpG islands in a gene body associated with oncogene overexpression where the enrichment of genes and hypermethylation of CpG islands in this model was similar to that in human HCC [60]. Therefore, besides recapitulating *MET* activation which is found in approximately 50% of HCC patients and strengthening several clinical trials using *MET* blocking agents, the study outcomes from this GEM integrated with those from human HCCs highlighted molecular similarities that may be useful to explore new mechanisms and to test new therapeutic interventions.

The *TGF-β*-activated kinase 1 (*TAK1*) belongs to the family of mitogen-activated protein kinase kinase kinases (*MAP3Ks*) [61]. It is activated by toll like receptors (TLRs), interleukin 1 (IL-1) receptor, tumor necrosis factor (TNF) receptor 1, and transforming growth factor beta (TGFβ) receptors [62], and in turn stimulates activation of *IKK-NF-κB* and *JNK* in hepatocytes, which are crucial regulators of cell survival, proliferation, and tumorigenesis, as well as lipid metabolism and insulin sensitivity [63]. Upon TNF or lipopolysaccharides (LPS) stimulation, *TAK1* plays an important role in the activation of *NF-κB* and prevents cancer through preventing Caspase-3-dependent hepatocyte and cholangiocyte apoptosis [64]. In hepatocytes of *TAK1* KO mice, the *NF-κB* pathway is inactivated which results in a functional gain of IKK-subunit *NF-kB* essential modulator (NEMO). The functional gain of NEMO is associated with necrosis, dysplasia of hepatocytes, biliary ductopenia, and early HCC development in 6- to 7-month-old *TAK1* KO livers. *TAK1* can also regulate AMPK activity where AMPK activation results in inhibition of *mTORC1*, a protein kinase complex that regulates lipid biosynthesis, cellular proliferation, and autophagy. Inactivation of *TAK1* decreases AMPK activation which consequently increase *mTOR* activity resulting in deregulation of autophagy [65,66,67].

Major urinary protein (MUP)-urokinase-type plasminogen activator (uPA) mice are uPA transgenic mice under the control of the mature hepatocyte-specific promoter for MUP [68]. MUP-uPA mice express high amounts of uPA specifically in the hepatocytes which lead to chronic ER stress. When MUP-uPA mice were placed on a HFD starting at six weeks of age, steatohepatitis very similar to pathology of human NASH eventually leads to spontaneous development of HCC at 40 weeks of age. The development of HCC was dependent on TNF production by inflammatory liver macrophages and TNF receptor 1 (TNFR1)-IκB kinaseb (IKKβ) signaling in hepatocytes [69]. 

Both MUP-uPA and STAM models mimic NASH-induced HCC but MUP-uPA tumors appear as “adenoma like” while the STAM model rapidly develops HCC. TAK1 and MUP-uPA models develop spontaneous tumors after long-term chronic liver damage recapitulating mutational processes in human HCC of TCGA [22].

## 3. Genomic Similarities among Mouse Liver Cancer Models

**Figure 2:**
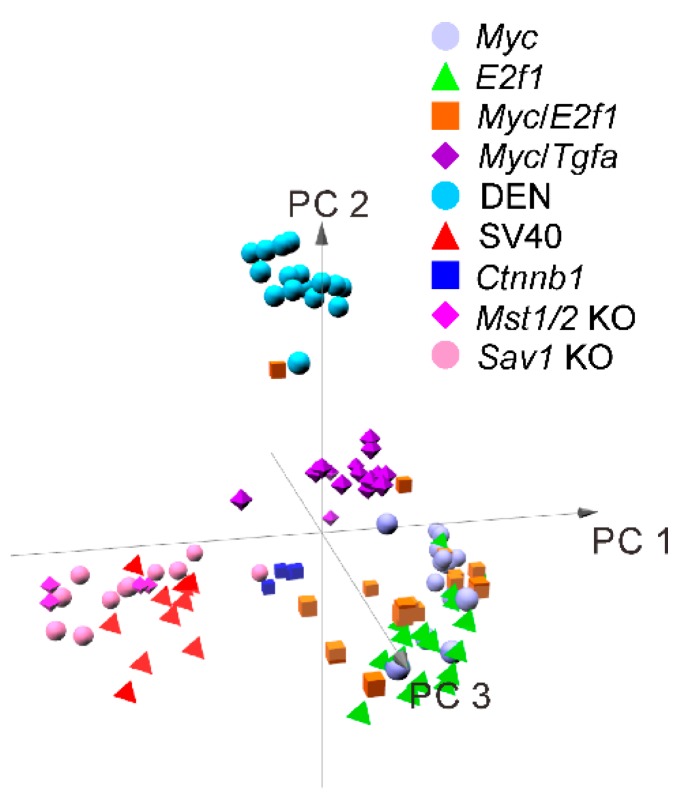
Cluster analysis of genetically engineered mouse model gene expression shows interrelationship among tumors with similar gene expression.

Hierarchical cluster analysis using gene expression data of nine different mouse models (*Myc*, *Myc/Tgfa*, *E2f1*, *Myc/E2f1*, *SV40*, *Mst1/2 KO*, *Sav1 KO*, *SV40* and *Ctnnb1*) from a recent study as mentioned above revealed that the DEN-induced model formed a tight cluster with distinct gene expression patterns reflecting that chemically induced models are different from those generated in genetically engineered mouse models (Figure 2) [15]. Tumors from *Mst 1/2* double-KO and *Sav1* KO mice clustered together as these genes are core regulator genes of the tumor suppressive Hippo pathway [15]. These tumors formed tight cluster with *SV40* tumor which were well separated from the rest of the mouse models suggesting that *SV40* has oncogenic activity mediated in part by suppression of the Hippo pathway or activation of its downstream target oncogene. In contrast, tumors from *Ctnnb1* clustered together with most tumors from the *Myc* models indicating that these two models share similar molecular characteristics in human HCC.

## 4. Genomic Resemblance

Genetically engineered mouse models recapitulate the complex multistep process of hepatocarcinogenesis so that researchers can understand it and design therapeutic experiments. However, human HCC is highly heterogeneous at molecular levels, enabling distinct and specific HCC mouse models to recapitulate only subsets of molecular events occurring in subgroups of HCC patients. To overcome this limitation, genomic studies have identified molecularly distinct subtypes of HCC with different clinical outcomes [13,54,70,71,72,73]. The establishment of the molecular and clinical resemblance of mouse cancer models to subtypes of human HCC subtypes will enable appropriate selection of mouse models for the investigations of the functional roles of newly discovered cancer genes and validation of potential therapeutic targets. There are several studies of systematic comparisons of human HCCs and mouse models at the molecular, genomic, and clinical levels [13,15,70,71]. 

In previous studies, gene expression signatures reflecting the clinical and molecular characteristics of tumors that are highly conserved in human and mouse tumors were reported [13,71]. In recent study, we reported genomic data from nine mouse HCC models, integrated and analyzed together with genomic data from human HCC to identify the mouse models that best resembled subtypes of human HCC and determine the clinical relevance of each model [15]. The resemblance of mouse models was classified based on prognostic, stem cell, and biological subtypes of human liver cancer as well as immune activity in mouse liver cancer models. 

Another study provided a comprehensive comparative genomic characteristic of four independent mouse models (*DEN*, *TAK1 KO*, *MUP-uPA*, and *STAM*) by systematically comparing somatic alteration data of human HCC derived from four human HCC cohorts to determine mouse–human similarities [22]. The following section will discuss based on these previous reported results.

### 4.1. Resemblance of Mouse Tumors to Prognostic Subtypes of Human Liver Cancer

*SV40*, *Mst1/2 KO*, *Sav1 KO*, and *Ctnnb1* mouse models best recapitulated the poor prognostic subtypes of human HCC that is high-proliferation and high-recurrence subtypes. In addition, these four mouse models were all classified to the cholangiocarcinoma subtype associated with poor prognosis. In contrast, the *Myc* model had the least aggressive phenotype while *E2f1*, *E2f1/Myc*, *E2f1/Tgfα*, and DEN-induced models were heterogeneous and were unequally split into poor and favorable prognoses. Interestingly, the highest metabolic activity was also observed in the most aggressive models, *SV40*, *Mst1/2 KO*, and *Sav1 KO* while tumors from less aggressive *Myc, E2f1*, and *DEN* models had the lowest glycolytic activity [15]. 

Another commonly used molecular classification, the Hoshida classification, divides HCCs into three subtypes S1, S2, and S3 where S1 and S2 subtypes have poor prognostic characteristics such as high cellular proliferation, stem-cell-like characteristics while S3 subtype is associated with better prognosis [74]. The silencing of Hippo (SOH) tumors (*Mst1/2 KO*, *Sav1 KO*) were associated with S1 subtype while most of the transgenic mouse were classified to S3 subtypes. 

A recent study that analyzed similarities between human HCC and mouse tumors, clustered human HCC, normal tissue, and mouse tumor using genomic data and classified the clusters from H1 to H3. The H1 subtype was enriched for low-grade tumors, low prevalence of *TP53* mutations, and had characteristics of Hoshida subtype 1 and 3 as well as TCGA iCluster 2 [5]. H2 tumors were enriched with low-proliferation genes while H3 was enriched with high-proliferation genes, high-grade tumors, and enrichment in *TP53* mutation. In addition, the H3 subtype was associated with Hoshida S1, S2 subtypes and TCGA iCluster 1 and 2 where iCluster 1 showed the worst prognosis among the three clusters. *TAK1* HCC model was similar to normal human liver samples while MUP-uPA model showed high correlation with H1 subtype, *Ctnnb1* model was associated with H2 subtype while STAM models were associated H3 subtype [22]. 

The *Alb-R26^Met^* model recapitulated the proliferative-progenitor subtype of human HCC, characterized by hypermethylation of CpG islands in the gene body associated with oncogene overexpression. The enrichment of genes and hypermethylation of CpG islands in *Alb-R26^Met^* tumors showed similar changes that were observed in human liver cancer [59,60].

### 4.2. Resemblance of Mouse Tumors to Stem Cell Subtypes of Human Liver Cancer

Mouse model resembling hepatic stem cell phenotype was catalogued using hepatic stem cell, epithelial cell adhesion molecule (EPCAM), and isocitrate dehydrogenase (IDH)-like signature [5]. Analysis of genomic signatures showed that only tumors from *Mst1/2* KO and *Sav1* KO models consistently reproduced hepatic stem cell features suggesting that inactivation of the Hippo pathway may lead to activation of stem cell characteristics in hepatocytes [15]. *Yap1* was reported to reprogram mature hepatocytes in adult mice into progenitor-like cells that could transdifferentiate into biliary epithelial cells [75]. EPCAM is frequently overexpressed in cancer-initiating cells in multiple cancer [76] while activating mutation of IDH blocks hepatic differentiation of hepatic stem cell though production of 2-hydroxyglutarate and suppression of the activity of *HNF4*, a master regulator of hepatic differentiation [77].

### 4.3. Resemblance of Mouse Tumors to Biological Subtypes of Human Liver Cancer

The β-catenin gene expression signature [BC or non-BC (NBC) subtype] previously defined as activation of β-catenin by somatic mutations in human HCC was used to identify tumors with activated β-catenin. The activation of β-catenin, which was observed in HCC patients with good prognosis [5], was observed in a majority of *Myc* transgenic mouse model in addition to *Ctnnb1* model while it was lowest in models associated with poor prognosis, *SV40*, *Mst1/2 KO*, and *Sav1 KO* models, suggesting β-catenin is a frequent co-activating partner of *Myc* in hepatocarcinogenesis. The DEN model showed a lack of β-catenin mutation as *Braf* and *Hras* are the most prevalent mutations in this model [78]. STAM mouse model showed *Ctnnb1* mutation at a rate comparable to human tumors and most closely recapitulated the molecular characteristics of human HCC. 

In addition to the *Ctnnb1* model, the *Myc* model was also classified as a BC subtype indicating that β-catenin is highly activated in *Myc* transgenic model while *SV40* and *Mst1/2 KO* models had the lowest β-catenin activity. The DEN model showed lack of β-catenin mutation as *Braf* and *Hras* are the most prevalent mutations in this model [78]. The STAM mouse model showed *Ctnnb1* mutation at a rate comparable to human tumors and recapitulated molecular characteristics of human HCC. 

Silencing of Hippo (SOH) pathway triggers activation of the oncogene *YAP1*, leading to development of HCC. SOH pathway along with high *Yap1* activity was observed in all *Mst1/2 KO*, and *Sav1 KO* models and in vast majority of *SV40* models [15]. Interestingly, tumors from the *Ctnnb1* model were classified as SOH subtype, clearly indicating an interaction between *Ctnnb1* and *Yap1* in the development of HCC while no other model had association with SOH subtype. 

### 4.4. Resemblance of Mouse Tumors to Human Immune Acitivity

Interferon gamma (IFNG) 6 scores that predict response to anti-PD-1 (pembrolizumab) therapy are derived from the average expression of the six genes (*Cxc110*, *Cxcl9*, *Ido1*, *Stat1*, *H2-Ea*, and *Ifng*) where higher mean expression values were associated with better response [79]. When tumors were dichotomized by mean IFNG6 score, the *E2f1* model was categorized to the high IFN6 group while models with silencing of Hippo pathway along with high *Yap1* activity such as *Mst1/2 KO* and *Sav1 KO* models as well as the majority of *SV40 and* DEN models were classified to the low IFN6 score group. In addition, the HCC models with high *Yap1* activity showed increased expression of checkpoint-inhibitory genes such as *Cd276* and *Pvrl2* while the DEN model had significantly lower expression of immune stimulatory genes such as *Cd86* compared to other models suggesting low immune activity in these models [15]. 

### 4.5. Resemblance of Mouse Tumors to Human Somatic Mutations and Pathway Alterations 

The DEN mouse model had heterogenous results for prognosis and had a high mutation rate with a unique mutation that has never been identified in human cancer and was unable to recapitulate the expression signature of any human tumors [15]. The DEN and STAM models showed much higher number of non-synonymous mutations and significantly mutated genes than TAK1 and MUP-uPA which far exceeded the average number of mutated genes in human. The STAM model had a pathway mutation rate comparable to those in humans for most pathways and despite low mutation rate of *TP53*, cell-cycle pathway alteration rate was similar to human tumors. Among DEN, TAK1, MUP-uPA, and STAM model, STAM was the only mouse model that recapitulated the molecular characteristics of human HCC [22].

## 5. Conclusions

The variety of mouse models has allowed researches to further understand the mechanism of hepatocarcinogenesis. With many options for mouse models, selecting the relevant mouse model that best recapitulates human HCC is important. In the post-genomic era, comprehensive genomic analysis has uncovered an unexpected complexity in the genetics of HCC that hampers a direct translation into mice using conventional transgenesis. The *Mst1/2* KO and *Sav1* KO models better mimicked the hepatic stem cell subtype that confers the poorest prognosis with lowest immune activity. Additionally, the STAM mouse model was molecularly similar to high-grade human HCC and tumors harboring *Ctnnb1* mutation, and the *Alb-R26^Met^* model with upregulated genes and hypermethylation of CpG islands was characterized by the poor prognosis human HCC subtype. The ability of mouse models to represent different prognosis, immunity, and histologic aggressiveness may provide a framework for guiding selection of the most appropriate mouse models for preclinical trials of novel drugs. 

## Figures and Tables

**Table 1:** Summary of mouse hepatocellular carcinoma (HCC) models with genomic resemblance to human HCCs.

**Carcinogen-Induced Model**	**Properties**	**Comments**
DEN	Intraperitoneal injection of diethylnitrosamine	DEN alone results in dysplastic nodules Additional stimulation lead to HCC progression No *TP53* and *CTNNB1* mutation Activation of the Ras/Raf/MEK/ERK signal transduction pathway
STAM	Combination of chemical and dietary intervention (HFD)	Mimic steatohepatitis Comparable to most of the human HCC pathways High *CTNNB1* mutation Associated with aggressive type (high-grade tumor and high proliferation) Low mutation rate of *TP53*
**GEM Model**	**Properties**	**Comments**
*MYC*	Albumin promoter	Long period of latency (20 months for HCC development) Co-activation with *E2F1* accelerates HCC development (9 months) Activation of *CTNNB1* Associated with good prognosis and low recurrence subtype of human HCC
*TGFα*	Metallothionein (MT) promoter	HCC development >12 months Co-activation with *MYC* accelerates HCC development (8 months) Rare *CTNNB1* mutation
*CTNNB1*	Intraperitoneal injection	Inactivation of Hippo pathway Associated with poor prognosis subtype of human HCC Associated with low immune activity
SV40 T	Antithrombin III promoter	Inactivation of Hippo pathway Associated with poor prognosis and high recurrence subtype of human HCC Stem-cell-like features Associated with low immune activity
*MST1/2* and *SAV1*	Genetic deletion	Inactivation of Hippo pathway Associated with poor prognosis and high-recurrence subtype of human HCC Stem-cell-like features Associated with low immune activity
*ARID1A*	Genetic deletion	At early stage, high expression promotes tumorigenesis At later stage, low expression promotes HCC progression
*TAK1*	Genetic deletion	Ductopenia, fibrosis, liver cell apoptosis, necrosis, hyperproliferation Histology: similar to normal human liver
*MUP-uPA*	uPA transgenic mouse with hepatocyte-specific promoter for MUP High-fat diet	Mimic steatohepatitis Histology: low-grade tumor, adenoma like Low mutation rate of *TP53*

DEN—diethylnitrosamine; HCC—hepatocellular carcinoma; STAM—Stelic Animal Model; GEM—genetically engineered mouse; *TGFα*—transforming growth factor α; *CTNNB1*—catenin beta 1; SV40 T—simian virus 40; ARID1A—AT-rich interaction domain 1A; *TAK1*—*TGF*-*β*-activated kinase 1; MUP—major urinary protein.
